# Blockade of ALDH in Cisplatin-Resistant Ovarian Cancer Stem Cells In Vitro Synergistically Enhances Chemotherapy-Induced Cell Death

**DOI:** 10.3390/curroncol29040229

**Published:** 2022-04-16

**Authors:** Fang Guo, Zhi Yang, Jalid Sehouli, Andreas M. Kaufmann

**Affiliations:** 1 Department of Gynecology, Shenzhen Hospital of Southern Medical University, Shenzhen 518100, China; guofang2200@smu.edu.cn; 2 Department of Orthopedics, Shenzhen Hospital of Southern Medical University, Shenzhen 518100, China; airboyyz@smu.edu.cn; 3 Department of Gynecology, Charité—Universitätsmedizin Berlin, Corporate Member of Freie Universität Berlin and Humboldt-Universität zu Berlin, Augustenburger Platz 1, 13353 Berlin, Germany; jalid.sehouli@charite.de; 4 HPV Research Laboratory, Department of Gynecology, Charité—Universitätsmedizin Berlin, Corporate Member of Freie Universität Berlin and Humboldt-Universität zu Berlin, Augustenburger Platz 1, 13353 Berlin, Germany

**Keywords:** chemotherapy resistance, regimens, apoptosis, tumor therapy, adjuvant therapy

## Abstract

Epithelial ovarian cancer (EOC) is the leading cause of gynecological cancer-related death. The high mortality and morbidity associated with EOC are mostly due to late diagnosis and chemotherapy drug resistance. Currently, the standard first-line chemotherapy regimen is systemic administration of platinum-based chemotherapy combined with a taxane. A major problem besides cisplatin resistance (occurring in nearly one-third of patients) is the greater toxicity of the drug combinations. A synergistic treatment with drug supporting activity could maximize the cytotoxic effects of chemotherapeutic agents on tumor cells while decreasing the dosage of each drug to potentially reduce toxicity. The ALDH-blocking agent Disulfiram (DSF), a clinically approved drug used for alcoholism treatment, has displayed promising anti-cancer activity. We previously described that blocking ALDH activity enhances the induction of apoptosis, especially in ovarian cancer stem cells treated with chemotherapeutic agents. In this study, we further investigated the synergistic effect of DSF in combination with cytotoxic chemotherapeutic drugs. The concentration of each chemotherapeutic agent could be significantly reduced with sustained efficacy on tumor cell apoptosis in cell lines in vitro (Dose-Reduction Index at IC_50_ from 1 to 50). Moreover, the potential chemo-sensitizing effects of DSF on ALDH-associated cisplatin-resistant ovarian cancer stem cells were also investigated and shown that in contrast to its high resistance to cisplatin, the cisplatin-resistant cells remain very sensitive to DSF-induced cytotoxicity (apoptosis and necrosis: cisplatin-resistant cells vs. parental cells: 60.4% vs. 20.5%). In combination with DSF and cisplatin, relatively more apoptosis and necrosis were induced in cisplatin-resistant cells than in their parental cells (apoptosis and necrosis: cisplatin-resistant cells vs. parental cells: 81.5% vs. 50.1%). A transcriptome analysis identified that ALDH was mainly enriched in the cancer-associated fibroblasts and showed that ALDH plays roles in responding to oxidative stress, metabolisms, and energy transition in the ALDH-associated cisplatin-resistant ovarian cancer stem cells. In conclusion, our data demonstrate a key role of ALDH-associated cisplatin-resistant cancer stem cells and identifies DSF as a potential adjuvant for a rational protocol design by computational quantitative assessment in vitro on ovarian cancer cell lines. Our work contributes to resolving the ALDH-associated cisplatin resistance and provides a resource for the development of novel chemotherapeutic regimens.

## 1. Introduction

Epithelial ovarian cancer (EOC) is a highly fatal gynecologic malignancy with over 150,000 deaths occurring worldwide each year [1]. The high mortality and morbidity associated with ovarian cancer are mostly due to late diagnosis and chemotherapy resistance [2]. Approximately 60% of women are diagnosed in an advanced stage that has already spread within the abdomen, and almost all will experience multiple recurrences and will eventually die due to chemotherapy resistance [3].

Currently, the standard first-line chemotherapy regimen in ovarian cancer is the systemic administration of platinum-based chemotherapy (cisplatin or carboplatin) combined with a taxane (paclitaxel or docetaxel) [4]. Platinum-based chemotherapy is the mainstay of treatment for ovarian cancer, and the major breakthrough in the last decade is the addition of paclitaxel [5]. A combination of platinum with paclitaxel showed higher therapeutic efficacy compared to platinum alone; however, due to the higher incidence of neurotoxicity in pretreated patients, its use is still limited [1]. It remains a priority to increase the sensitivity of tumor cells to cisplatin-based chemotherapy or to identify new regimens for antitumor adjuvant treatment.

Increasing evidence suggests that the existence of cancer stem cells (CSCs), which are characterized by unlimited self-renewal capacity and tumorigenicity, are the main culprit contributing to chemoresistance and tumor recurrence of ovarian cancer [3]. Several specific surface markers have been used to identify CSCs in ovarian cancer, such as CD44, c-kit, CD133, CD117, EpCAM, LGR5, and LY6A [6]. Moreover, stemness-associated transcription factors are elevated in CSCs, including Oct3/4, Sox2, and Nanog [7]. Although these markers have been employed for the isolation and characterization of ovarian CSCs from ovarian cancer cell lines, a major challenge here is targeting and overcoming the chemo-resistance property of CSCs, which is a serious bottleneck for the effective treatment of ovarian cancer.

Recently, the enzyme aldehyde dehydrogenase (ALDH) holds the attractive distinction among CSC markers as ALDH may be more than just a CSC marker but may have a potential functional role in CSC biology [8,9]. Studies have shown that ALDH enzyme expression and activity may be associated with particular cell types in ovarian tumor tissues and vary according to cellular states (proliferating or dormant) [6], indicating that ALDH isozymes may play essential roles in lineage differentiation and pathogenesis for ovarian cancer pathophysiology [10]. Our previous studies have found that ALDH+ cells display stem-like characteristics such as enhanced expression of stem cell transcription factors, clonogenicity, sustained proliferation, and resistance to chemotherapy [7]. We have also described that blocking ALDH activity by Disulfiram (DSF), which is the first-line drug for alcoholism behavioral therapy, enhanced the induction of apoptosis, especially in ovarian cancer stem cells.

In this study, we further investigated the synergistic effect of DSF in combination with cytotoxic chemotherapeutic drugs. Currently, no consistent conclusions about combination therapy effect have been made. This present study aimed to investigate the key role of ALDH-associated cisplatin-resistant cancer stem cells in mediating cisplatin resistance in human ovarian cancer cell lines and explore DSF as a potential adjuvant for a rational protocol design by computational quantitative assessment in vitro on ovarian cancer cell lines. 

## 2. Materials and Methods

### 2.1. Cell Lines and Cell Culture

The ovarian cancer cell lines SKOV3IP1 and IGROV1 (kindly provided by Dr. Hagen Kulbe) were cultured in RPMI-1640 medium (Invitrogen, Heidelberg, Germany) supplemented with 10% fetal bovine serum and 100 IU/mL penicillin/100 μg/mL streptomycin (both from Millipore, Darmstadt, Germany). A2780-cis-re and SKB-R3-cis-re are cisplatin-resistant cell lines derived from the parental cisplatin-sensitive cell lines of A2780 and SKB-R3, respectively, by selection with increasing concentrations of cisplatin from 0 µmol/L to 20 µmol/L with increments of 0.5 µmol/L every 72 h. After each selection, the media was removed and cells were allowed to recover for a further 72 h [11,12]. This development period was carried out for approximately 6 months, after which time IC_50_ concentrations were re-assessed in each resistant cell line. Cells were then maintained continuously in the presence of cisplatin at these new IC_50_ concentrations for a further 6 months. All cells were maintained in a humidified atmosphere at 37 °C and 5% CO_2_ and regularly screened for mycoplasma contamination.

### 2.2. Drug Sensitivity Measuring by MTT Assay

Cells were seeded in 96-well plates at a density of 4000 cells per well in 100 μL drug-free medium and incubated overnight. Then, cells were exposed to chemotherapeutic drugs, cisplatin alone or DSF alone or a DSF/cisplatin combination, at indicated concentrations in fresh medium for a further 72 h. Cells without any treatment were used as control. The MTT assay (Thermo Fisher Scientific, Darmstadt, Germany) was performed according to the suppliers’ protocol after 72 h incubation. Absorbance OD_590nm_ was measured by a Bio-Rad microplate reader (BD Bioscience, Heidelberg, Germany).

The relative viability (%) of cells was calculated by the following formula: relative viability (%) = (absorbance of sample/absorbance of control) × 100% [6]. Cells cultured for 72 h without any drug treatment were used as control. Each concentration was in triplicate, and the assay was repeated independently three times.

### 2.3. Clonogenicity Assay

Cells were exposed to cisplatin (3 μM and 6 μM for SKB-R3 and SKB-R3-cis-re cell lines, 4 μM and 8 μM for A2780 and A2780-cis-re cell lines) for 24 h. The cisplatin-treated cells were then collected and cultured in a drug-free medium in a 6-well plate at a density of 2000 cells/well for 7–10 days. Fixation and staining of colonies were done by adding 2–3 mL of a mixture of 6.0% glutaraldehyde and 0.5% crystal violet. Colonies of at least 50 cells were counted and compared with non-treated cells as controls.

### 2.4. Flow Cytometric Analysis of ALDH Activity

ALDH activity was assessed using the ALDEFLUOR kit (Stem Cell Technologies, Durham, NC, USA). Briefly, cells (4 × 10^4^/well) were incubated with ALDH substrate BAAA for 30 min at 37 °C following the manufacturer’s instructions. Cells treated with diethyl-aminobenzaldehyde (DEAB), a specific ALDH inhibitor, were used as a control to establish the baseline fluorescence and define the cut-off for ALDEFLUOR-positive cells.

### 2.5. Flow Cytometric Analysis of Cell Cycle 

Analysis of cell cycle progression and detection of apoptosis was performed using flow cytometric analysis of DNA staining. All drug-treated and untreated cells were harvested by trypsinization. Cells (4 × 10^4^/well) were suspended in 100 μL PBS and fixed in 900 μL 70% ethanol overnight. The cells were then incubated with RNase (100 μg/mL, Sigma, Darmstadt, Germany) and propidium iodide (50 μg/mL, Sigma, Darmstadt, Germany) for 30 min. The data from 10,000 cells for each sample were acquired by FACS Scan (BD Bioscience, Heidelberg, Germany) and DNA content and cell cycle distribution were analyzed.

### 2.6. Flow Cytometric Analysis of Cellular Apoptosis

The apoptotic status was determined by flow cytometry with FLUOS-conjugated Annexin-V and propidium iodide Kit (Roche, Mannheim, Germany). Cells (4 × 10^4^/well) were incubated in a 24-well plate overnight and treated with 5 μM cisplatin alone or 1 μM DSF alone or in combination for a further 72 h. All cells were then harvested and suspended in 100 μL binding buffer containing FITC-conjugated Annexin-V (2 µL)/PI (2 µL) and incubated at RT for 15 min in the dark. Apoptosis and necrosis were evaluated measuring FL3 (PI) and FL1 (Annexin-V) in events gated to single cells by FSC/SSC by FACS analysis. Viable cells were Annexin-V−/PI−, early apoptotic cells were Annexin-V+/PI−, late apoptotic cells were Annexin-V+/PI+ and necrotic cells were Annexin-V-/PI+. Early and late apoptosis was combined considering total apoptosis.

### 2.7. Two- and Three-Drug Combination Treatment

Cells were seeded in 96-well plates and incubated overnight, followed by treatment with various concentrations of DSF, cisplatin, and paclitaxel. For each cell line, the range of dosage of the drug treatment was selected to cover the concentrations below and above the IC_50_ values of each drug. The combination of the drugs was designed at a constant ratio which was at the IC_50_ concentration of each of the drugs used so that the contribution of the effect by each drug to the combination would be equal [13]. Cells were treated with every single drug or every two-drug combination, or all three drugs in combination for 72 h and then were subjected to MTT assay.

### 2.8. Combination Effect Analyses

Description of synergism or antagonism in drug combination studies was based on the CompuSyn software of Chou and Martin [13,14]. Briefly, the combination index (CI) value in a combination is a quantitative measurement of the degree of drug interactions in synergism and antagonism at a given measurement effect. CI < 1, =1, and >1 indicate synergism, additive effect, and antagonism, respectively [14]. The smaller the CI value is, the stronger the synergistic effect is. The dose-reduction index (DRI) value is a measurement of how many times the dose of each drug in a synergistic combination may be reduced at a given effect level when compared with the doses of each drug alone [14].

### 2.9. Further Verification of DSF Potentiation of Chemotherapeutic Drug Sensitivity

To further verify the capability of DSF in potentiating chemotherapy drug sensitivity, ovarian cancer cell lines were treated with each conventional chemotherapeutic agent, cisplatin and paclitaxel, or their combination in conjunction with or without DSF. The dose of cisplatin and paclitaxel was selected at the original IC_50_ concentration of each drug for each cell line, as well as the dose after reduction according to the quantitative combination measurement. The dose for DSF in conjunction was the specific IC_50_ value for each cell line. Untreated cells were used as a control in all experiments.

### 2.10. Patient Selection

The ovarian cancer patient was pathologically diagnosed and treated by cisplatin-based chemotherapy. Written informed consent was obtained from the patient and this study received ethical approval from the ethical committee of Baoan Maternal and Children Health Hospital (LLSC 2020-03-28).

### 2.11. Droplet-Based scRNA-Seq

Single-cell RNA sequencing libraries were created using the Chromium Single Cell 3’ Library, Gel Bead & Multiplex kit, and chip kit (10X Genomics) aiming for 5000 cells per library according to manufacturer instructions. All cells were treated with the same master mix and in the same reaction vessel. This droplet-based system uses barcodes (1 for each cell) and unique molecular identifiers (UMIs, 1 for each unique transcript) to obtain a unique 3′-mRNA gene expression profile from every captured cell. All samples were sequenced by the Illumina HiSeq4000 and mapped to the human reference genome (GRCh38) by Cell Ranger (10X Genomics).

### 2.12. Statistical Analysis

Statistical analyses were performed using GraphPad Prism 5. Quantitative analysis was performed with CompuSyn software. Differences were considered statistically significant at *p* < 0.05. All data presented were representative of three independent experiments.

## 3. Results

### 3.1. Quantitative Analysis of Enhanced Cell Line Sensitivity to Two- and Three-Drug Combinations

Our previous studies demonstrated that ALDH is overexpressed in vitro in spheroid-derived cells (SDC), due to enrichment of cancer stem-like cells, which were more resistant to cisplatin treatment with higher IC_50_ when compared to monolayer-derived cells (MDC), representing bulk cancer cell populations [6,15]. Cell sorting was done to get ALDH+ cells, and ALDH+ cells display stem-like characteristics, such as sustained proliferation capability and resistance to chemotherapy [7]. We also demonstrated that Disulfiram (DSF), a drug used for alcoholism treatment, displayed an inhibitory effect on ALDH enzyme activity and showed cytotoxic effects on ovarian cancer cell lines due to enhanced ROS induction and subsequently leads to a loss of ALDH-mediated protection against oxidative stress [7]. Moreover, DSF sensitized ovarian cancer cells to cisplatin treatment and enhanced cisplatin-induced apoptosis, both in SDC and MDC.

In the present studies, we have used a quantitative method to determine synergism or antagonism of DSF in chemotherapy drug treatment. Three pairs of two-drug combinations and a three-drug combination were tested in vitro on established ovarian cancer cell lines and cisplatin-resistant derivatives. The combination index (CI) and dose-reduction index (DRI) values at different effect levels are presented for SKOV3IP1 (Table 1) and for IGROV1 (Table 2) cells. The results showed that cisplatin + DSF and cisplatin + paclitaxel + DSF exhibited superior synergistic effects at broad effect level ranges from IC_50_ to IC_90_ in both cell lines. The combination of paclitaxel and DSF yielded the greatest synergism at high effect levels of IC_90_ in the SKOV3IP1 cell line, while they showed an antagonistic effect in the IGROV1 cell line. However, cisplatin + paclitaxel + DSF combination still showed a desirable synergistic effect in the IGROV1 cell line (Table 2). Furthermore, due to synergistic effects, the dosage of each drug may be reduced even by a hundredfold while maintaining equal anti-tumor cell toxicity once they are combined. The combination of three drugs continued to yield synergistic effects, while DRI tended to be even higher than in two-drug combinations which was expected. These data support that DSF sensitizes ovarian cancer cells to cisplatin and paclitaxel treatment.

### 3.2. Verification of Chemotherapy Drug Effect Potentiation by DSF

**Figure 1:**
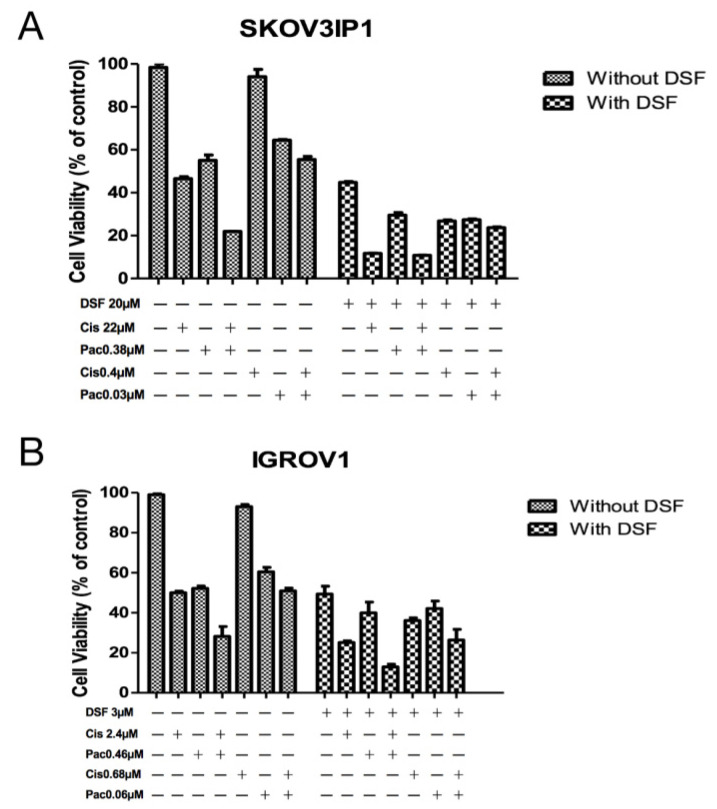
Verification of enhancement of chemotherapy drug effect by DSF. SKOV3IP1 cells (**A**) and IGROV1 cells (**B**) were treated at indicated drug concentration or drug combinations for 72 h. Cellular viability was detected by MTT assay. Untreated cells were used as control. Cis: cisplatin. Pac: paclitaxel. DSF: disulfiram. All data presented are representative of three independent experiments.

To verify the potentiation in drug combinations, we further treated the cells at the original IC_50_ concentration and at reduced IC_50_ concentrations calculated according to DRI for 72 h. As shown in Figure 1, for cell line SKOV3IP1, the IC_50_ of cisplatin is at 22 µM; the IC_50_ of paclitaxel is at 0.38 µM, and the combination of these two drugs at their original IC_50_ concentration without DSF addition further increased cytotoxicity. According to Table 1, the combination of cisplatin at the concentration of 0.4 µM, which is a reduction by 51.06-fold from 22 µM, with paclitaxel at 0.03 µM, which is a reduction by 11.89-fold from 0.38 µM, achieved the same cytotoxic effect with a cellular viability of around 50% (Figure 1A). Either paclitaxel alone or cisplatin alone at the concentration of reduced concentrations only exhibited slight cellular proliferation inhibitory effect by MTT assay. However, DSF significantly reduced the cellular viability when IC_50_ of DSF at 20 µM was added. Importantly, the combination with DSF and reduced concentration of chemotherapeutic drugs reached almost the same effect as cisplatin + paclitaxel combination at their IC_50_ (cell viability is around 20%). A similar effect was observed on cell line IGROV1. The combination of cisplatin at 0.68 µM, which is a reduction by 3.5-fold from 2.4 µM, with paclitaxel at 0.06 µM, which is a reduction by 7.4-fold from 0.46 µM, induced the same cytotoxic effect as with cisplatin at 2.4 µM alone or paclitaxel at 0.46 µM alone. In conjunction with DSF at its IC_50_ at 3 µM for this cell line yielded very strong synergistic effects with the conventional anti-tumor therapeutic drugs (Figure 1B).

### 3.3. Characterization of Cisplatin-Resistant Cell Lines for Proliferation and ALDH Expression

**Figure 2:**
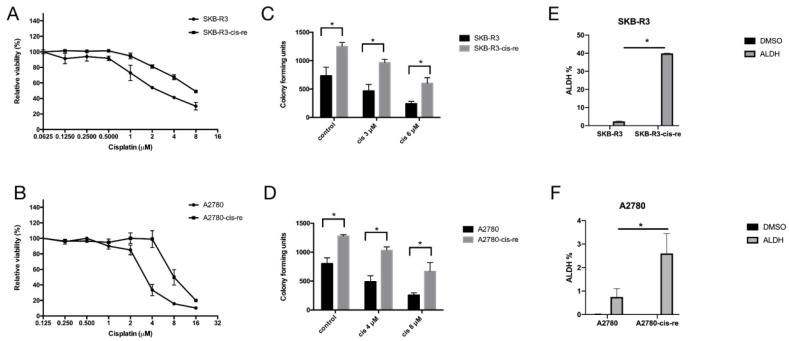
Cell lines SKB-R3-cis-re and A2780-cis-re are more resistant to cisplatin-induced apoptosis and contain a larger ALDH+ cell pool. (**A**,**B**) MTT assay. The cisplatin-resistant cells and their parental cells were exposed to different concentrations of cisplatin for 72 h and viability was related to untreated control. (**C**,**D**) Clonogenicity assay. Cells exposed to indicated concentrations of cisplatin for 24 h were cultured in a drug-free medium in six-well plates at a cell density of 2000 cells per well for 7–10 days. The colonies with ≥50 cells were counted. (**E**,**F**) Graphical representation of the statistical analysis of ALDH activity in cisplatin-resistant cells and their parental cells (*n* = 3). Cells treated with diethylamino-benzaldehyde (DEAB), which is a specific ALDH inhibitor, were used as a control. Numbers represent ALDH+ cells (%). One representative of three independent experiments is shown (* *p* < 0.05).

In order to characterize the cisplatin-resistant cells, we selected cisplatin-resistant cell lines SKB-R3-cis-re and A2780-cis-re from parental cell lines SKB-R3 and A2780 by several rounds of continuous culture in a medium containing progressively increased concentrations of cisplatin. First, the cytotoxic effect of cisplatin on both resistant cell lines and the parental cell lines was compared by MTT assay (Figure 2A,B and Table 3). The SKB-R3 cells were sensitive to cisplatin with an IC_50_ at 72 h of treatment of 2.87 ± 0.12 µM. In contrast, the SKB-R3-cis-re cell line was resistant to cisplatin at approximately threefold higher concentrations of SKB-R3 with an IC_50_ at 72 h of treatment of 7.38 ± 0.03 µM. The A2780-cis-re cells are also more resistant to cisplatin with an IC_50_ after 72 h of treatment to 8.64 ± 0.51 µM compared to parental A2780 cell with an IC_50_ after 72 h of 3.39 ± 1.05 µM. Next, a clonogenicity assay was performed to determine cell survival and stemness after drug treatment. As shown in Figure 2C,D, the colony number in the cisplatin-resistant cell lines was increased approximately twofold as compared to their parental cell lines. These results confirm the increased resistance, the cellular reproduction capacity, and stemness of cisplatin-resistant cells.

To further explore the cell line stemness, i.e., cancer stem-like cell frequency in the cell lines, we performed an ALDEFLUOR assay that detects ALDH-positive cells that have stem cell features, as we have shown before [7,16]. Our results showed that in comparison with the parental cell lines, the cisplatin-resistant cell lines contained higher numbers of an ALDH+ population. Cell lines SKB-R3-cis-re vs. SKB-R3 had 39.85% vs. 2.355% ALDH+ cells, respectively (*p* < 0.05), while the cell lines A2780-cis-re vs. A2780 had a proportion of 2.6% vs. 0.75% ALDH+ cells (*p* < 0.05) (Figure 2E,F).

**Figure 3:**
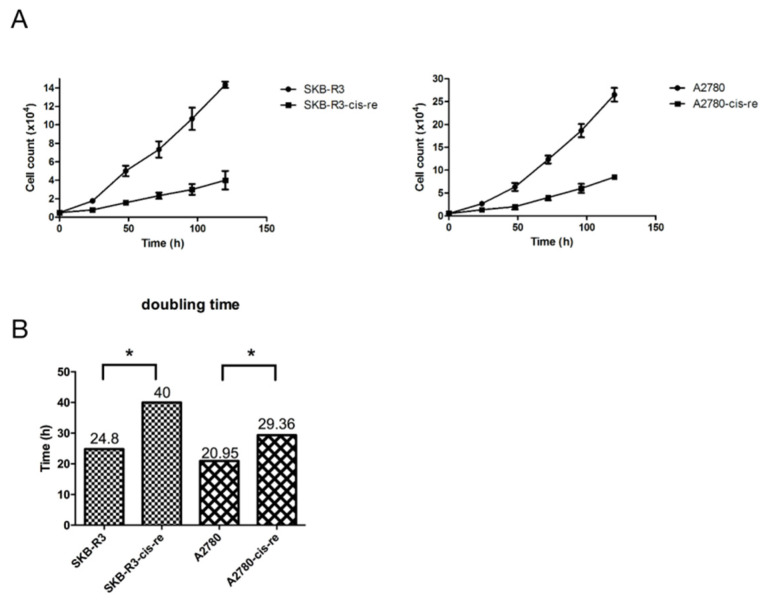
Cisplatin-resistant cells have a lower proliferation rate. (**A**) Growth curves of cisplatin-resistant cells and their parental cells. (**B**) The doubling time (h) is presented. Mean and s.d. of three experiments (* *p* < 0.05).

**Figure 4:**
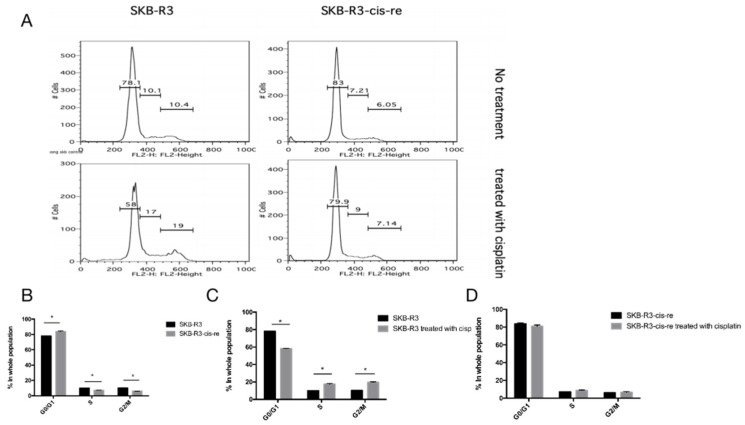
Cell cycle analysis in SKB-R3. (**A**) Histogram of flow cytometric DNA content analysis. (**B**–**D**) Cell cycle parameters in SKB-R3-cis-re and SKB-R3 cells. Mean and s.d. of three experiments (* *p* < 0.05).

**Figure 5:**
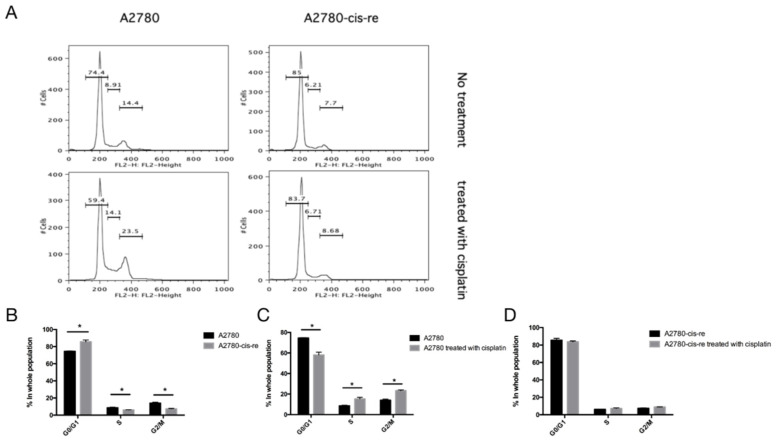
Cell cycle analysis in A2780. (**A**) Histogram of flow cytometric DNA content analysis. (**B**–**D**) Cell cycle parameters in A2780-cis-re and A2780 cells. Mean and s.d. of three experiments (* *p* < 0.05).

In the cell culture, the cisplatin-resistant cells grow markedly slower than the parental cells. We compared the doubling time and cell cycle parameters in these two cell lines. Figure 3A shows the growth curves of both cell lines. The doubling time of SKB-R3-cis-re cells (40 h) is significantly longer than that of the parental cells (24.8 h, *p* < 0.05). Likewise, the doubling time of A2780-cis-re cells (29.36 h) is also significantly longer than that of A2780 cells (20.95 h, *p* < 0.05) (Figure 3A,B). Flow cytometry analysis of the cell cycle indicated that in comparison to the parental cell lines, the cisplatin-resistant cell lines had significantly higher G0/G1 and lower S-phase and G2-phase populations. After cisplatin treatment (concentration of IC_50_ for 72 h), the population of G0/G1 phase increased, while S-phase and G2-phase population decreased significantly in SKB-R3 and A2780 cells (*p* < 0.05) (Figure 4 and Figure 5). These data are in agreement with results from other labs supporting that cisplatin displays its cytotoxic effects on cancer cell lines by arresting the cell cycle in the G2/M phase. However, our results showed that no significant cell cycle changes were induced by cisplatin treatment in SKB-R3-cis-re and A2780-cis-re cells at the same cisplatin concentration.

### 3.4. Disulfiram Is Highly Cytotoxic in Cisplatin-Resistant Cells

**Figure 6:**
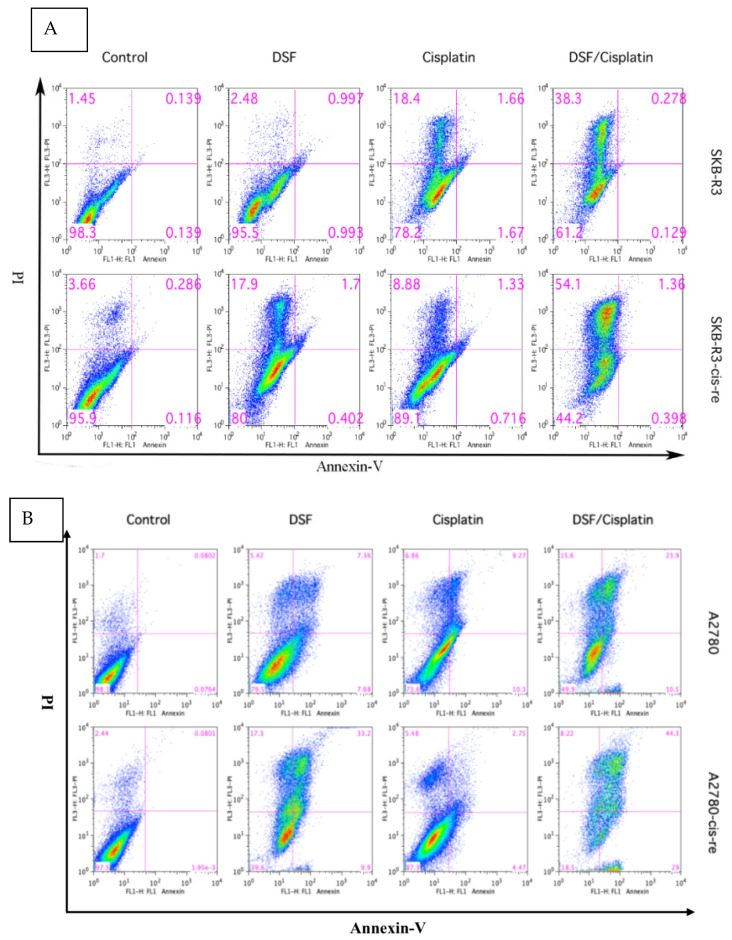
Disulfiram is highly cytotoxic in cisplatin-resistant cells and induces more apoptosis combined with cisplatin. Apoptotic status was determined in SKBR3 (**A**) and A2780 (**B**) cell lines by FLUOS-conjugated Annexin-V and propidium iodide Kit (Roche, Mannheim, Germany) using flow cytometry following the manufacturer’s instructions. Cells were treated with cisplatin alone or DSF alone or their combination for 72 h. LL, LR, UR, and UL are representative of live cells, early apoptotic cells, late apoptotic cells, and necrotic cells, respectively. One representative of three independent experiments is shown.

We have shown that inhibiting ALDH with DSF sensitizes ovarian cancer cells to cisplatin treatment. The cisplatin-resistant cell lines demonstrated a higher frequency of ALDH+ cells. Thus, we further assessed the effect of DSF on cisplatin-resistant cells. The cell viability and the cellular apoptotic status were tested after treatment with DSF alone (1 μM) or cisplatin alone (SKB-R3-cis-re and SKB-R3: 1 μM; A2780-cis-re and A2780: 2.5 μM) or in combination. The results showed that compared with SKB-R3 cells, the SKB-R3-cis-re cells were more resistant to the cisplatin treatment at the same concentration. Interestingly, the SKB-R3-cis-re cells were also more sensitive to DSF, with more apoptotic status cells identified after DSF treatment at the same concentration. Figure 6 shows that exposure to either DSF or cisplatin alone for 72 h only slightly reduced the cellular viability. However, a significant decrease of cellular viability was induced by the drug combinations compared to individual treatment with DSF or cisplatin (*p* < 0.01) at these concentrations. The results showed that in the SKB-R3 cell line apoptosis and necrosis increased from 21.8% to 38.8% in cisplatin-treated cells and cells treated with cisplatin combined with DSF, respectively. However, a significant increase in cellular apoptosis and necrosis was induced by SKB-R3-cis-re cells when the cells were treated with the combination compared to cisplatin treatment alone, with apoptosis and necrosis amounting to 55.8% from 10.9% by cisplatin alone. Similar results were observed in the A2780 and A2780-cis-re cell lines. In A2780 cells, apoptosis and necrosis increased from 26.4% in cisplatin-treated cells to 50.1% in the drug combination-treated cells. In A2780-cis-re cells, apoptosis and necrosis increased from 12.7% in cisplatin-treated cells to 81.5% in the drug combination-treated cells. These data indicated that DSF sensitizes for cisplatin treatment and suppresses cell viability by inducing more apoptosis and necrosis both in cisplatin-resistant cells and their parental cells.

### 3.5. ALDH+ Cells were Relatively More Resistant to Chemotherapeutics

**Figure 7:**
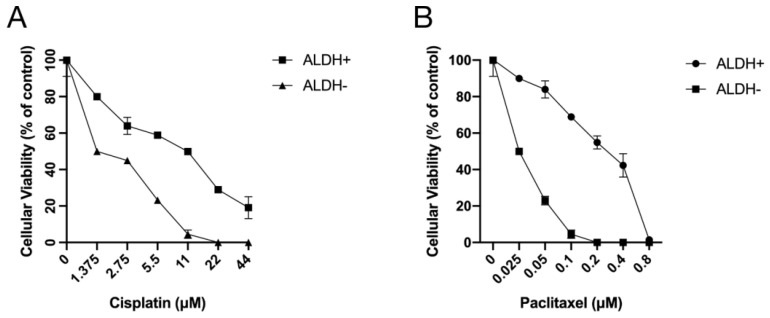
ALDH+ cells were relatively more resistant to treatment with chemotherapeutics. ALDH+/− cells sorted from A2780-cis-re cells were treated with (**A**) cisplatin or (**B**) paclitaxel at indicated concentrations for 72 h. Cellular viability was detected by MTT assay.

Next, ALDH+ and ALDH− cells which were FACS-sorted from A2780-cis-re cells were then treated with cisplatin (0–44 μM) or paclitaxel (0–0.8 μM) for 72 h and subjected to MTT assay. As shown in Figure 7, ALDH+ cells were more resistant to cisplatin or paclitaxel treatment compared to ALDH− cells at any concentration investigated. The relative cellular viability was significantly higher in ALDH+ cells than ALDH− cells after cisplatin or paclitaxel treatment at the same concentration (*p* < 0.05). These results suggested that ALDH+ cells were relatively more resistant to chemotherapeutics which also indicated the vital roles of ALDH when cells respond to cisplatin treatments.

### 3.6. scRNA-Seq Analysis of High-Grade Serous Ovarian Cancer

**Figure 8:**
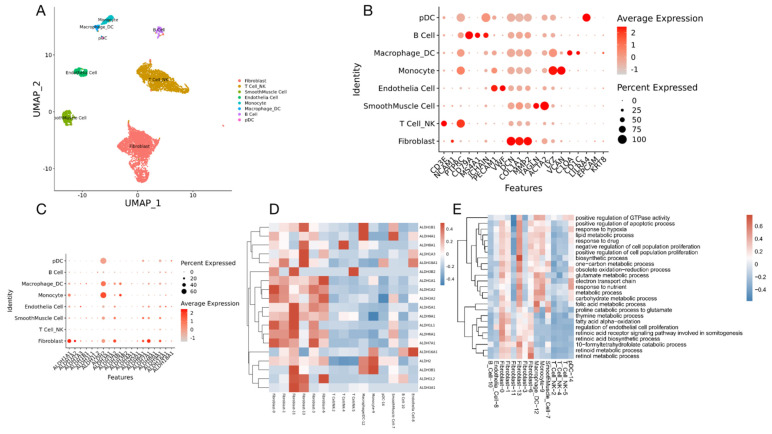
scRNA-Seq analysis of a high-grade serous ovarian cancer. (**A**) UMAP representation of all single cells color-coded for their assigned major cell type. (**B**) The expression of the marker genes used for the cell type annotation is indicated on the DotPlot. (**C**) DotPlot visualization of ALDH genes listing on scRNA-seq clusters. Cell types are listed on the y-axis, and ALDH genes are listed along the x-axis. Dot size reflects the percentage of cells in a cluster expressing each gene; dot color reflects expression level (as indicated in legend). (**D**) Heat map showing scaled expression of ALDH genes among different cell types. (**E**) GO terms showing all pathways including ALDH genes.

A 46-year-old multiparous woman with regular menstrual cycles presented with dyspepsia and abdominal distension. The imaging and test results suggested primary ovarian cancer. The patient underwent one post-neoadjuvant chemotherapy and surgery. The final dignosis was high grade serous ovarian cancer (HGSOC). We collected scRNA-seq data and obtained 18,403 cells with high quality transcriptomic data. After normalization, principal component analysis (PCA) was performed using 16,236 variably expressed genes to assign all cells to different clusters. Cells were divided into 15 clusters representing 8 major cell types based on canonical marker gene expression across these clusters: T cells/NK cell (clusters 2,4,5, marked by CD3D, CD3E, NCAM1, PTPRC), B cells (cluster 10 marked by MS4A1, JCHAIN), endothelial cells (cluster 8 marked by PECAM1, VWF), cancer-associated fibroblasts (clusters 0,1,3,6,11,13, marked by DCN, COL1A1, MMP2), smooth muscle cells (cluster 7 marked by TAGLN, ACTA2), monocytes (cluster 9 marked by LYZ, VCAN), macrophages (cluster 12 marked by C1QA, CD1C), and dendritic cells (cluster 14 marked by LILRA4, JCHAIN) (Figure 8A,B).

Since we have found that ALDH+ cells were relatively more resistant to chemotherapeutics, we tried to further identify which cell clusters were enriched for the ALDH gene transcripts. Interestingly, the results showed that the ALDH was mainly enriched in the cancer-associated fibroblasts (Figure 8C,D). To further explore the roles of ALDH, the pathways including ALDH genes were selected for gene ontology (GO) studies. The GO analysis indicated that several biological processes are involved, including regulation of the apoptotic process, response to hypoxia, electron transport chain, metabolic process, and several others (Figure 8E). These data suggest that in addition to altered oxidative stress, which we have shown in our previous findings [16], ALDH activity may also influence cellular proliferation and apoptotic regulation, metabolism, and energy transition.

## 4. Discussion

Platinum-based chemotherapy with the substitution of paclitaxel is the most commonly used combination for treating advanced-stage EOC after surgery [17,18]. Unfortunately, cancer cells either intrinsically are or relatively rapidly become resistant to cisplatin-based chemotherapy, leading to relapse and therapeutic failure [1]. Another major problem besides cisplatin resistance is the greater toxicity of the combination. Cisplatin induces DNA damage through inter-strand or intra-strand cross-linking of DNA [19]. Its dose-limiting toxicity is nephrotoxicity, peripheral nerve toxicity, and toxicity to the cochlea (the inner ear toxicity) [20]. Paclitaxel is a microtubule poison that arrests cells in mitosis [21]. It binds along the length of microtubules thereby stabilizing and suppressing the normal cell division [22,23]. Its dose-limiting toxicity is hypersensitivity, neutropenia, and peripheral neuropathy [5,24]. Both drugs suppress cell dynamics, leading to mitotic arrest and apoptosis in dividing cells. Their toxic effects are only partially overlapping. For an enhanced treatment efficacy, efforts should be made to maximize the cytotoxic effects of chemotherapeutic agents on tumor cells while minimizing their toxic effects on normal cells [25].

The aldehyde dehydrogenase (ALDH) superfamily comprises 19 isozymes that catalyze the oxidation of aldehydes. Landen and colleagues first identified ALDH1A1+ cells possessing CSC phenotype in ovarian cancer cell lines. However, these cells can become re-sensitized to chemotherapy by ALDH1A1 silencing using nanoliposomal siRNA in ovarian cancer cell line SKOV3TRip2 and A2780cp20 [26]. Our previous results are in agreement with results from other labs supporting that ALDH+ cells display stem-like characteristics such as enhanced expression of stem cell transcription factors, clonogenicity, sustained proliferation, and resistance to chemotherapy [7,10,16].

DSF exerts its action as a drug for alcoholism behavioral treatment by blocking ALDH enzymes, leading to the accumulation of acetaldehyde as an intermediate of alcohol detoxification. Accumulation of acetaldehyde leads to discomfort. However, this blockage also disrupts a scavenger effect for reactive oxygen species (ROS) in cells sensitizing them for apoptosis. Therefore, DSF has proven strong anti-cancer activity [16,27]. Our previous studies further demonstrated that DSF itself exhibits dose-dependent cytotoxicity in ovarian cancer cell lines and the ALDH activity was significantly inhibited by DSF. Importantly, a suppressive effect of DSF on stemness of ALDH+ CSCs, as shown by inhibition in a sphere formation assay, was shown. Our previous studies also confirmed that blocking ALDH activity by DSF enhances induction of apoptosis, especially in ovarian cancer stem cells [7].

In this study, we found that DSF effectively sensitized cancer cells to cisplatin treatment. In cultured ovarian cancer cells, treatment with DSF potentiated the combination effect with cisplatin and paclitaxel even at its lower dose, and significantly enhanced cisplatin-induced apoptosis. Due to its chemo-sensitizing effects, DSF is very promising to be combined with cisplatin-based chemotherapy to improve the therapeutic outcome. 

Further, quantitative assessment of DSF combinations in vitro could help to design rational protocols for adjuvant chemotherapy in ovarian cancer. We have used a quantitative method to determine synergism or antagonism of DSF in chemotherapy drug treatment. Due to synergistic effects, the dosage of each drug may be reduced even by a hundredfold while maintaining equal anti-tumor cell toxicity once they are combined. In this study, our results showed that DSF yielded superior synergistic effects combined with cisplatin alone as well as in a cisplatin/paclitaxel/DSF three-drug combination. Importantly, the combination with DSF and reduced concentration of chemotherapeutic drugs reached almost the same effect as cisplatin + paclitaxel combination at their IC_50_. This DSF synergistic effect in multiple drug combinations provides potential therapeutic benefits. Firstly, it could increase or at least maintain the same efficacy but decrease the dosage of each drug to potentially reduce toxicity [14]. Secondly, for cisplatin-resistant patients, DSF could increase the efficacy of therapeutic effects by potentially sensitizing cancer cells to cisplatin treatment. Thirdly, co-application could minimize or slow down the development of drug resistance in patients under first or second-line therapy [28].

As previously reported, effective cancer treatment has to not only destroy cancer cells that represent the bulk of the tumor cell population but also destroy cisplatin-resistant cells in CSCs [29]. We found that the cisplatin-resistant cells have a significantly lower proliferation rate and longer doubling time with a higher proportion of cells blocked in the G0/G1 phase. It has been known for a long time that conventional anticancer agents primarily target cycling cancer cells [30]. The quiescent cancer cell population located in the G0/G1 phase is resistant to chemotherapeutic agents. Cisplatin can only target the cycling and proliferating cells. Similarly, paclitaxel is predominately an M-phase-specific drug that stabilizes microtubules causing an M-phase arrest followed by apoptosis [31]. Therefore, all of these drugs may lose their anticancer activity if the cancer cells are prevented from entering the cell cycle by G0/G1-phase arrest. Importantly, our results showed that the cisplatin-resistant cell lines contained higher numbers of an ALDH+ population. DSF is a very efficacious ALDH inhibitor and CSC-targeting agent. Thus, we further assessed the effect of DSF on cisplatin-resistant cells. Our results show that in contrast to its high resistance to cisplatin, the cisplatin-resistant cells remain very sensitive to DSF-induced cytotoxicity, indicating strong chemoresistance-reversing activity of DSF. In combination with DSF and cisplatin, more apoptosis and necrosis were induced in cisplatin-resistant cells than in their parental cells. These data confirmed the potential chemo-sensitizing effects of DSF on ALDH-associated cisplatin-resistant ovarian cancer stem cells.

Through the scRNA-seq analysis from one high-grade serous ovarian cancer (HGSOC) patient, we identified that the ALDH was mainly enriched in the cancer-associated fibroblasts. Our transcriptome analysis also identified the distribution of different ALDH genes in cancer-associated cell clusters. The GO analysis of ALDH genes indicated that several biological processes are involved, including regulation of the apoptotic process and response to oxidative stress which are in alignment with our previous findings [7]. The results also showed that metabolism and energy transition may correlate with ALDH and play critical roles in the ALDH-associated cisplatin-resistant ovarian cancer stem cells and indicate that targeting ALDH may be an approach for overcoming therapeutic resistance.

Moreover, due to its synergistic effect in combination with cytotoxic chemotherapeutic drugs, the doses of each chemotherapeutic agent could be reduced with sustained efficacy on tumor cell apoptosis, thereby potentially reducing the toxicity while maintaining efficacy. Our present quantitative data analyses also provide evidence for a rational protocol design in a clinical study. Further investigation of the biochemical and cell biologic mechanisms of this synergistic effect is still needed.

## Figures and Tables

**Table 1:** Two- and three-drug combination effect at 50%, 75%, and 95% inhibition of SKOV3IP1 cell growth.

Drug Combination	Combination Index at			Dose-Reduction Index at		
	IC_50_	IC_75_	IC_90_	IC_50_	IC_75_	IC_90_
Cis + Pac	0.104	0.267	3.170	51.06 11.89	4.02 54.81	0.32 252.58
Cis + DSF	0.123	0.176	0.311	11.74 37.36	4.80 156.08	1.97 652.05
Pac + DSF	1.021	0.048	0.004	1.05 14.36	29.81 70.96	845.61 350.68
Cis + Pac + DSF	0.286	0.110	0.196	49.80 11.60 158.48	5.39 73.59 175.17	1 466.87 193.61

**Table 2:** Two- and three-drug combination effect at 50%, 75%, and 95% inhibition of IGROV1 cell growth.

Drug Combination	Combination Index at			Dose-Reduction Index at		
	IC_50_	IC_75_	IC_90_	IC_50_	IC_75_	IC_90_
Cis + Pac	0.42	0.36	0.36	3.50 7.38	3.23 18.88	2.97 48.29
Cis + DSF	0.24	0.33	0.52	5.09 25.38	3.15 57.17	1.95 128.76
Pac + DSF	2.99	24.49	202.63	0.47 1.12	0.05 0.17	0.006 0.02
Cis + Pac + DSF	0.32	0.19	0.16	5.28 11.12 26.30	6.24 36.49 113.13	7.38 119.78 486.71

Cis: cisplatin. Pac: paclitaxel. DSF: Disulfiram. CI: combination index. DRI: dose-reduction index.

**Table 3:** The IC_50_ values of parental and cisplatin resistant cell lines.

Cell Line	IC_50_ (uM)
SKB-R3	2.87 ± 0.72
SKB-R3-cis-re	7.38 ± 1.23
A2780	3.39 ± 1.12
A2780-cis-re	8.64 ± 2.73

## Data Availability

The data presented in this study are available on request from the corresponding author.
